# Beyond cell replacement: restoring organelle homeostasis as the next frontier of regenerative medicine

**DOI:** 10.3389/fbioe.2026.1953752

**Published:** 2026-10-13

**Authors:** Filipa Ferreira-Antunes, Marta F. Lima, Anabela Moreira, Fernando J. Silva, Belém Sampaio-Marques, António J. Salgado

**Affiliations:** 1 School of Medicine, Life and Health Sciences Research Institute (ICVS), University of Minho, Braga, Portugal; 2 ICVS/3B’s, PT Government Associate Laboratory, Guimarães, Portugal; 3 Department of Pharmacology, UCL School of Pharmacy, London, United Kingdom; 4 UCL Centre for Nerve Engineering, London, United Kingdom

**Keywords:** EVS, neurodegenerative disorders, organelle homeostasis, secretome, stem cells

## Abstract

Neurodegenerative diseases (NDs) have traditionally been viewed as disorders driven by individual pathological processes, including protein aggregation, mitochondrial dysfunction, oxidative stress, endoplasmic reticulum (ER) stress, impaired autophagy, and neuroinflammation. However, growing evidence demonstrates that these mechanisms are not isolated events but interconnected manifestations of disrupted intracellular organelle communication. Dynamic interactions among mitochondria, the ER, lysosomes, peroxisomes, and other organelles coordinate calcium signaling, bioenergetics, proteostasis, lipid metabolism, and cellular stress adaptation, thereby preserving neuronal homeostasis. Progressive disruption of this communication network represents a fundamental driver of neuronal dysfunction and degeneration, suggesting that restoration of organelle homeostasis may constitute a more effective therapeutic strategy than targeting individual molecular pathways. In parallel, regenerative medicine has evolved beyond the concept of cell replacement toward approaches that enhance the resilience and repair capacity of surviving cells. Among these, mesenchymal stem cell (MSC)-derived secretomes and extracellular vesicles (EVs) have emerged as promising cell-free therapeutics owing to their diverse repertoire of proteins, lipids, metabolites, cytokines, growth factors, and regulatory RNAs capable of simultaneously modulating multiple cellular pathways. Accumulating evidence indicates that these bioactive cargos modulate mitochondrial function, alleviate ER stress, improve autophagy-lysosomal activity, reduce oxidative stress and neuroinflammation, and reinforce proteostasis, collectively promoting intracellular homeostasis. Here, we propose organelle homeostasis as a promising focus for regenerative medicine and discuss how cell-free therapies may redefine therapeutic strategies for NDs by restoring the intracellular communication networks that sustain cellular resilience. Finally, we highlight current translational challenges and future opportunities for engineering next-generation cell-free therapeutics targeting complex diseases.

## 1 Introduction

With the steady rise in global life expectancy, neurodegenerative disorders (NDs) have emerged as one of the most pressing public health challenges of the 21st century. Traditionally characterized by the progressive loss of neuronal structure and function in both the central and peripheral nervous systems, the underlying etiology of NDs is incredibly complex (Aranda-Abreu et al., 2026; Singh et al., 2019; Teleanu et al., 2022). In fact, it involves a highly interconnected network of dysregulated cellular pathways, ranging from chronic neuroinflammation and oxidative stress to mitochondrial dysfunction and protein misfolding, which current therapies fail to arrest or reverse (Aranda-Abreu et al., 2026; Dugger and Dickson, 2017; Fraldi et al., 2016; Nixon and Rubinsztein, 2024; Picca et al., 2020).

To address this therapeutic gap, regenerative medicine has focused on stem cell-based therapies over the last few decades. While pioneering strategies primarily aimed at cell replacement, the field has undergone a major paradigm shift. Emerging evidence reveals that the primary therapeutic efficacy of stem cells is largely mediated by their potent paracrine activity (Assunção Silva et al., 2022; Assunção-Silva et al., 2018; da Silva et al., 2023; Da Silva et al., 2025; Daneshmandi et al., 2020; Drago et al., 2013). Consequently, the use of stem cell-derived products, either as a complex mixture (i.e., the secretome) or as isolated, targeted components (i.e., extracellular vesicles (EVs) and specific microRNAs), has gained significant attention as a strategy to restore physiological homeostasis, particularly in NDs.

In this mini-review, we explore recent advances in approaches focused on stem cell-derived secretome and its components as a cell-free therapeutic platform for NDs. Specifically, we highlight the cellular mechanisms and discuss the powerful synergistic effects that make them a promising frontier in regenerative medicine of the central nervous system (CNS).

## 2 Neurodegeneration as a disease of organelle network dysfunction

For decades, research on NDs has focused on neuronal cell loss and protein aggregation as defining endpoints. However, neuronal loss appears to be the outcome of a more diffuse, earlier breakdown in intracellular systems that maintain neuronal homeostasis (Aranda-Abreu et al., 2026; Picca et al., 2020; Teleanu et al., 2022; Trigo et al., 2022; Udayar et al., 2022; Yuan et al., 2024). Dysfunction in these systems can be detected years prior to the onset of neurodegeneration and clinical symptoms, showing that when neurodegeneration appears, the main pathological process has started long before. In this section, we will give an overview of impaired pathways that are now being targeted as a driver of neurodegeneration in several neurological conditions (Figure 1).

**FIGURE 1:**
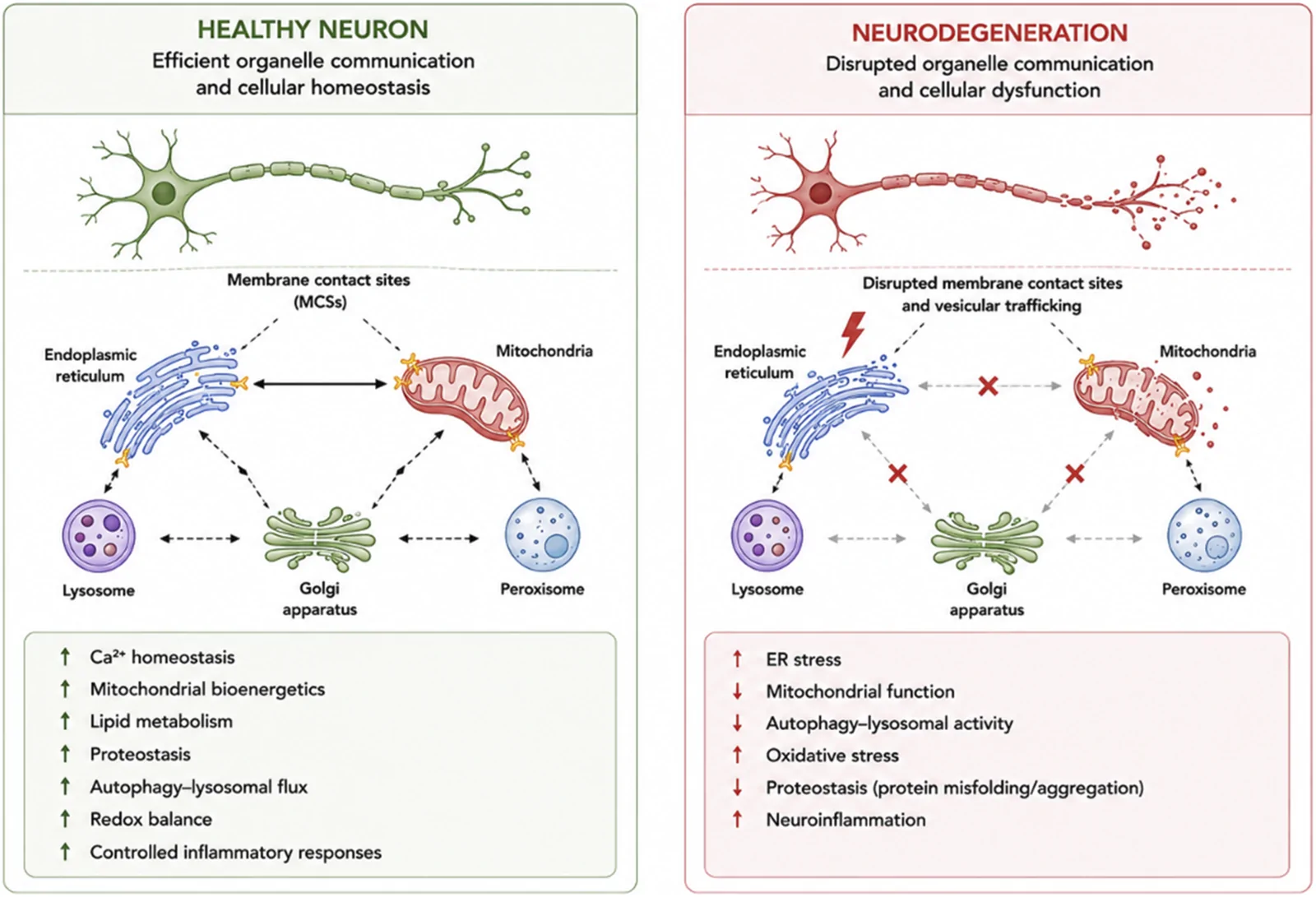
Organelle communication network in healthy neurons and its disruption during neurodegeneration. Under physiological conditions (left), membrane contact sites (MCSs) and vesicular trafficking coordinate communication between the endoplasmic reticulum (ER), mitochondria, lysosomes, the Golgi apparatus, and peroxisomes, maintaining Ca^2+^ homeostasis, mitochondrial bioenergetics, lipid metabolism, proteostasis, autophagy–lysosomal flux, redox balance, and controlled inflammatory responses. During neurodegeneration (right), disruption of organelle communication impairs inter-organelle crosstalk, leading to ER stress, mitochondrial dysfunction, defective autophagy–lysosomal activity, oxidative stress, proteostasis collapse, and neuroinflammation, ultimately contributing to protein aggregation, neuronal dysfunction, and progressive neurodegeneration. Created with BioRender.com.

Mitochondrial dysfunction and oxidative stress have long been explored as major drivers of neurodegeneration in several NDs such as Parkinson’s Disease (PD), Alzheimer’s Disease (AD) and Huntington’s Disease (HD) (Aranda-Abreu et al., 2026; Barazzuol et al., 2021; Markaki and Tavernarakis, 2020; Moradi Vastegani et al., 2023; Picca et al., 2020; Trigo et al., 2022; Zong et al., 2024). Mitochondria, having evolved from a bacterial ancestor, have a unique genomic material known as mitochondrial DNA (mtDNA), capable of self-replication (Picca et al., 2020; Trigo et al., 2022; Zong et al., 2024). Mitochondria are recognized as having a central function in generating ATP via oxidative phosphorylation, mediating lipid metabolism, Ca^2+^ homeostasis, and apoptosis (Aranda-Abreu et al., 2026; Singh et al., 2019; Trigo et al., 2022; Zong et al., 2024). Different factors can contribute to mitochondria dysfunction, such as elevated reactive oxygen species (ROS) levels, mtDNA mutations and impaired mitophagy, which ultimately results in reduced ATP production, imbalance in Ca^2+^ levels, and, eventually, apoptosis (Moradi Vastegani et al., 2023; Picca et al., 2020; Singh et al., 2019; Trigo et al., 2022; Zong et al., 2024). In neuronal cells this is particularly relevant because neurons are high energy demanding cells, making them especially vulnerable to mitochondrial dysfunction (Singh et al., 2019; Trigo et al., 2022; Zong et al., 2024). Oxidative stress happens when there is an imbalance between ROS production and antioxidant defenses (Aranda-Abreu et al., 2026; Moradi Vastegani et al., 2023; Picca et al., 2020; Singh et al., 2019; Teleanu et al., 2022). This condition may arise from excessive ROS generation caused by mitochondrial, endoplasmatic reticulum (ER) or peroxisomal dysfunction, impaired antioxidants defenses, including glutathione, superoxide dismutase (SOD), catalase (CAT), or, more often, a combination of these processes (Allen et al., 2012; Alnasser, 2025; Bjørklund et al., 2021; Ježek et al., 2018; Singh et al., 2019; Trigo et al., 2022). ROS damage lipids (membrane peroxidation), proteins (oxidative modification) and DNA (for example, mtDNA). Since neurons have high oxygen consumption, high lipid contents and relatively limited antioxidant capacity, they are especially vulnerable to oxidative stress.

ER is the primary site of protein folding, intracellular Ca^2+^ storage, and protein quality control, ensuring that only properly folded and functional proteins are transported through the secretory pathway (Aranda-Abreu et al., 2026; Chen et al., 2023; Yuan et al., 2024). These quality control mechanisms are essential for maintaining cellular proteostasis. However, when the protein-folding capacity of the ER is exceeded, misfolded and unfolded proteins accumulate within the ER lumen, triggering ER stress and activation of the unfolded protein response (UPR) (Aranda-Abreu et al., 2026; Yuan et al., 2024). Initially, the UPR acts as an adaptive mechanism that modulates ER homeostasis by transiently attenuating protein synthesis, upregulating molecular chaperones, and remodeling gene expression to enhance protein folding and degradation (Aranda-Abreu et al., 2026; Chen et al., 2023; Kim et al., 2018; Rabouille, 2017; Yuan et al., 2024). Nevertheless, when ER stress is prolonged or unresolved, the UPR shifts toward pro-apoptotic signaling, ultimately contributing to neuronal dysfunction and cell death (Chen et al., 2023; Kim et al., 2018; Yuan et al., 2024). Consistent with the exceptionally high secretory and metabolic demands of neurons, ER stress is now recognized as an early pathological event in several NDs, suggesting that impaired ER proteostasis contributes to disease initiation and progression.

Lysosomes are multifunctional organelles that serve as the primary degradative compartment of the cell while also playing essential roles in maintaining cellular homeostasis and viability (Aranda-Abreu et al., 2026; Jain and Zoncu, 2022; Rabouille, 2017; Wang and Yang, 2024). Beyond their degradative function, lysosomes act as central signaling hubs that regulate diverse processes, including nutrient sensing, membrane repair, energy metabolism, immune responses, and autophagy (Fraldi et al., 2016; Nixon and Rubinsztein, 2024; Picca et al., 2020; Udayar et al., 2022). Efficient lysosomal function depends on coordinated autophagosome-lysosome fusion, proper luminal acidification, and tightly regulated Ca^2+^ signaling (Nixon and Rubinsztein, 2024; Picca et al., 2020; Udayar et al., 2022). Disruption of any of these processes compromises lysosomal degradative capacity, resulting in the accumulation of damaged organelles and misfolded proteins. Consequently, lysosomal dysfunction contributes to proteostasis collapse, impaired organelle quality control and chronic cellular stress, all of which are recognized as central features of NDs.

Proteostasis refers to the cellular network that regulates protein synthesis, folding, trafficking, conformational maintenance and degradation to preserve proteome integrity and function (Chen et al., 2023; Hartl et al., 2011; Santra et al., 2019). With aging and disease, the capacity of the proteostasis network progressively declines, leading to the accumulation and aggregation of misfolded proteins (Chen et al., 2023; Chowdhury et al., 2026; Hartl et al., 2011; Santra et al., 2019). These aggregates can become toxic and propagate between cells, meaning that the pathology is not exclusive to the cell of origin (Santra et al., 2019). Consequently, dysfunction of molecular chaperones, the ubiquitin-proteasome system, autophagy-lysosomal pathways and stress response mechanisms collectively drives a self-perpetuating cycle of proteostasis collapse and proteotoxicity. The interplay between the CNS immune system and neurons has also received increased attention in the development of NDs (Aranda-Abreu et al., 2026; Muzio et al., 2021; Picca et al., 2020). Neuroinflammation in the CNS is mainly mediated by microglia and astrocytes, which, under homeostasis, have protective roles such as clearing debris and responding to injury. In NDs, microglia and astrocyte activation can become chronic, leading to the release of pro-inflammatory cytokines (e.g., TNF-α, IL-1β) and ROS (Garcia et al., 2024; Kaur et al., 2019; Muzio et al., 2021; Picca et al., 2020). This creates a cycle in which neuronal damage triggers more glial activation, which causes even more damage. As a result, neurons are constantly exposed to a hostile extracellular environment.

Despite substantial differences in etiology and pathological progression, these neurological disorders share several interconnected cellular vulnerabilities, including mitochondrial dysfunction, ER stress, impaired proteostasis, and lysosomal/autophagic dysregulation, as described in Table 1. Importantly, however, the contribution of each pathway and its relationship with disease-specific molecular drivers varies considerably between disorders. Additionally, emerging paradigms in cellular biology reveal that these pathologies do not occur separately. Instead, neurodegeneration is the result of multiple dysregulations of a highly integrated physical and biochemical communication network (Aranda-Abreu et al., 2026; Dugger and Dickson, 2017; Jain and Zoncu, 2022; Trigo et al., 2022). Within this intracellular network, organelles do not merely coexist but actively communicate through transient and permanent membrane contact sites (MCSs) to coordinate cellular metabolism, calcium dynamics, and proteostasis (Barazzuol et al., 2021; Chowdhury et al., 2026; Jain and Zoncu, 2022). These processes allow organelles to function and adapt in response to shifting cellular environments and maintain homeostasis. Due to their high energetic demand and limited regenerative capacity, neurons require a high metabolic efficiency signaling and inter-organelle communication (Bjørklund et al., 2021; Dugger and Dickson, 2017; Moradi Vastegani et al., 2023). With aging, this communication is impaired and usually evolves into neurodegenerative processes (Aranda-Abreu et al., 2026; Dugger and Dickson, 2017; Fernandes et al., 2023). In the next section, we will provide a brief resume of how organelle homeostasis is affected in NDs and the relevance of restoring organelle homeostasis as the next frontier in regenerative medicine.

**TABLE 1:** Disease-specific molecular and organelle-related abnormalities across neurological disorders.

Disease	Disease context	Major organelle/pathway abnormalities	Disease-specific molecular features	Main affected cells/processes
Alzheimer’s disease (AD) (Lane et al., 2018)	Chronic, progressive neurodegeneration	Mitochondrial dysfunction, ER stress, impaired lysosomal/autophagic function, altered ER–mitochondria contacts	Aβ and tau pathology, altered lipid metabolism, MAM-associated signaling	Neurons, astrocytes, microglia; synaptic dysfunction and neuronal degeneration
Parkinson’s disease (PD) (Beitz, 2014)	Chronic, progressive neurodegeneration	Mitochondrial dysfunction, impaired mitophagy, ER stress, lysosomal/autophagic dysfunction	α-Synuclein pathology, PINK1/Parkin-dependent mitophagy, LRRK2-associated pathways	SNpc dopaminergic neurons, astrocytes, microglia; synaptic and axonal dysfunction
Huntington’s disease (HD) (Tong et al., 2024)	Chronic, progressive neurodegeneration	Mitochondrial dysfunction, impaired autophagy, ER stress, altered proteostasis	Mutant huntingtin, transcriptional dysregulation, altered axonal transport and neuronal metabolism	Striatal medium spiny neurons and cortical neurons; synaptic and axonal dysfunction
Amyotrophic lateral sclerosis (ALS) (Rizea et al., 2024)	Chronic, progressive neurodegeneration	ER stress, mitochondrial dysfunction, impaired proteostasis/autophagy, defective axonal transport	TDP-43, SOD1, FUS, C9orf72-associated mechanisms	Motor neurons, astrocytes, microglia; axonal degeneration and neuromuscular dysfunction
Multiple sclerosis (MS) (Correale et al., 2017)	Chronic immune-mediated CNS disease with inflammatory and progressive phases	Mitochondrial dysfunction, oxidative stress, ER stress, lysosomal/autophagic alterations	Immune dysregulation, demyelination, oligodendrocyte injury, axonal degeneration	Oligodendrocytes, neurons/axons, microglia, astrocytes and infiltrating immune cells
Spinal cord injury (SCI) (Silva et al., 2014)	Acute injury followed by secondary damage and chronic remodeling	Mitochondrial damage, ER stress, oxidative stress, altered autophagy, excitotoxicity	Secondary injury, neuroinflammation, excitotoxicity, BBB disruption, glial scar formation	Neurons, oligodendrocytes, astrocytes, microglia and infiltrating immune cells
Ischemic stroke (Feske, 2021)	Acute vascular injury followed by secondary injury and repair	Mitochondrial failure, ER stress, oxidative stress, excitotoxicity, altered autophagy	Ischemia/reperfusion injury, excitotoxicity, BBB disruption, neuroinflammation	Neurons, endothelial cells, astrocytes, microglia; vascular and synaptic dysfunction

## 3 Organelle homeostasis: a new therapeutic target for regenerative medicine

Inter-organelle communication occurs through multiple mechanisms, including vesicular trafficking and MCSs such as mitochondria-associated ER membranes (MAMs), mitochondria-lysosome interfaces and peroxisome-ER junctions (Barazzuol et al., 2021; Chen et al., 2023; Chowdhury et al., 2026; Fernandes et al., 2023; Wang and Yang, 2024; Yuan et al., 2024) (Figure 1). Neuronal homeostasis relies heavily on proper ER function and its interaction with other organelles (Chen et al., 2023; Wang and Yang, 2024; Yuan et al., 2024). MCSs are regions where membranes of two organelles are within 10–30 nm of each other (Wang and Yang, 2024). Although they exist in various organelles, they are mostly present in the ER, due to its ability to extend throughout the cell and its high lipid biosynthesis, requiring lipid transport domains, usually present in MCSs (Chen et al., 2023; Chowdhury et al., 2026; Wang and Yang, 2024; Yuan et al., 2024). A major class of ER-organelle contact sites is mediated by vesicle-associated membrane protein (VAMP)-associated proteins (VAPs), which function as ER-resident tethering proteins (Wang and Yang, 2024). Various lipid transport proteins (LTPs) bind to ER-localized VAPs and are responsible for the transport of lipids. Mutations in LTPs are associated with a variety of diseases and are a good example of the effects of poor organelle or damaged organelle communication effects on NDs. Mutations in VPS13, a group of glycerolipid transfer proteins that bind to ER-associated VAPs and are located in lysosomal membranes, result in mitochondrial dysfunction associated with PD (Chen et al., 2023; Chowdhury et al., 2026; Jain and Zoncu, 2022; Wang and Yang, 2024; Yuan et al., 2024). In fact, ER-mitochondria MCSs alterations have been observed in PD and AD patients and have been a proposed target with relevant therapeutic value for amyotrophic lateral sclerosis (ALS) and frontotemporal dementia (FTD) (Chen et al., 2023; Fernandes et al., 2023; Jain and Zoncu, 2022; Kim et al., 2018; Rabouille, 2017; Wang and Yang, 2024; Yuan et al., 2024).

ER-Golgi communication is mediated primarily through vesicles rather than MCSs. With aging, cellular proteostasis and secretory activity are progressively impaired and affect the ER-Golgi efficiency (Chowdhury et al., 2026; Jain and Zoncu, 2022). With defects in protein control and increased burden of misfolded proteins in the ER, the Golgi apparatus is compromised and defects in Golgi trafficking also contribute to an increased accumulation of misfolded proteins in the ER, highlighting their interdependence (Chowdhury et al., 2026). Major neurological disorders such as AD and PD are highly related with misfolded protein aggregation (Dugger and Dickson, 2017). Whenever the tension in the ER exceeds due to accumulation of misfolded proteins, it leads to apoptosis induction in neurons. More specifically, the IP3 isoform of ER receptor donates calcium to the mitochondria, resulting in neuronal cell death (Chen et al., 2023; Wang and Yang, 2024; Yuan et al., 2024) and highlighting the intercommunication between ER, Golgi and mitochondria.

Previously, we mentioned mitochondrial dysfunction as a driver of neurodegeneration and aging. Indeed, mitochondria are responsible for maintaining a wide range of processes such as the cellular redox balance, calcium buffering and iron-sulfur cluster biogenesis, and amino acid metabolism and fatty acid oxidation. In neurons, mitochondria signaling is complex, and a structured and well-regulated mitochondrial signaling and function is needed to ensure proper activity (Aranda-Abreu et al., 2026; Barazzuol et al., 2021; Ježek et al., 2018; Markaki and Tavernarakis, 2020; Picca et al., 2020; Singh et al., 2019; Trigo et al., 2022). To accomplish this, these processes require a regulated exchange of substrates and products with other compartments in the cell, including the ER, cytosol, lysosomes and peroxisomes. The mitochondria can establish contact sites with various organelles through mitochondria-derived vesicles (MDVs) and MAMs (Barazzuol et al., 2021; Fernandes et al., 2023). Lately, MAMs have indeed emerged as a central hub involved in different fundamental cell processes, such as the ER stress response, mitochondrial and ER dynamics, inflammation, apoptosis, and autophagy (Barazzuol et al., 2021; Fernandes et al., 2023; Trigo et al., 2022). Consistently, dysregulation of ER-mitochondria crosstalk has been associated with different pathological conditions. For instance, the ER is the site of intracellular Ca^2+^ storages, and Ca^2+^ flux is controlled in MAMs (Jain and Zoncu, 2022; Barazzuol et al., 2021). Specifically, some lipid species regulate the architecture of these membrane systems. Deficiency in the lysosomal hydrolase B-galactosidase leads to cellular buildup, which accumulates within MAMs and leads to increased Ca^2+^ flux into the mitochondria, resulting in increased apoptosis (Sano et al., 2009; Barazzuol et al., 2021).

The inositol 1,4,5-triphosphate receptor (IP3R), an intracellular Ca^2+^ channel protein, connects to voltage-dependent anion channels (VDAC) through the binding action of glucose-regulated protein 75 (GRP75), forming the IP_3_R-GRP75-VDAC complex, which is involved in Ca^2+^ transport from the ER (Chen et al., 2023; Jain and Zoncu, 2022; Wang and Yang, 2024). In AD, increases in MAM functions and ER-mitochondrial communications were found in fibroblasts from patients with familial and sporadic AD (Wang and Yang, 2024). Recently, it has been proposed that MAM plays a role in amyloid β production and Tau dysfunction (Wang and Yang, 2024). Interestingly, α-synuclein (asyn), a major protein misfolded in PD, is present in MAMs (Jain and Zoncu, 2022; Rabouille, 2017; Wang and Yang, 2024). In cells overexpressing asyn, it was found that asyn interferes with the IP_3_R-GRP75-VDAC complex, reducing ER-mitochondria contacts by diminishing MAM activity and ultimately contributing for Ca^2+^ imbalances, often described as a major contributor for neurodegeneration (Barazzuol et al., 2021; Chowdhury et al., 2026). Loss of function of the protein DJ-1 is also heavily linked with a familial form of PD. DJ-1 interacts with the IP_3_R-GRP75-VDAC complex and its absence or malfunction leads to impaired ER-mitochondrial communication, causing mitochondrial dysfunction (Barazzuol et al., 2021; Chowdhury et al., 2026; Antipova and Bandopadhyay, 2017; Neves et al., 2022). VAPB is an integral ER membrane protein that interacts with the protein tyrosine phosphatase interacting protein-51 (PTPIP51) in the MAM (Wang and Yang, 2024). This interaction is involved in the regulation of Ca^2+^ transport, ATP production and autophagy and is usually formed in the synaptic region, and its interaction is stimulated by neuronal activity. Deletion of either VAPB or PTPIP51 causes morphology changes and impairs synaptic function. Moreover, reduced expression of these proteins was observed in patients with terminal AD, suggesting their involvement in the pathology progression as well as in ALS and FTD (Barazzuol et al., 2021; Chowdhury et al., 2026; Wang and Yang, 2024).

Mitochondria-lysosome contact sites mediate calcium, phosphatidylinositol-4-phosphate (PI(4)P), cholesterol and iron transfer from the lysosome to the mitochondria (Dagda and Das Banerjee, 2015; Jain and Zoncu, 2022; Singh et al., 2019). Mitochondria and lysosomes communicate through the formation of contacts promoted by active, GTPase-activating protein TBC1D15 and GTP-bound Ras-related lysosomal protein RAB7, possibly due to the role of RAB7 in vesicle trafficking. In fact, several lysosomal storage disorders lead to dysregulation of mitochondria-lysosome contacts and mitochondria defects are also associated with lysosomal impairments. In genetic forms of PD linked to mutant variants of *GBA1* (which encodes a lysosomal membrane protein), there is an increased mitochondria-lysosome interplay associated with an impaired TBC1D15-mediated transport.

Importantly, signaling pathways are major players in regulating intra-organelle homeostasis. For instance, the mechanistic target of rapamycin complex 1 (mTORC1) responds to a wide variety of cues such as nutrients, growth factors and ATP (Chowdhury et al., 2026; Jain and Zoncu, 2022; Wang and Yang, 2024). Autophagy initiation and lysosome biogenesis are inhibited via mTORC1-dependend phosphorylation of Unc-51 related kinases (ULK) and transcription factors EB (TFEB) and E3 (TFE3) (Chowdhury et al., 2026; Jain and Zoncu, 2022; Guo et al., 2018; Ren et al., 2020; Zhu et al., 2019). In turn, lysosome also plays a role in modulating mTORC1 signaling whether by the effect of a lysosome-localized AMP-activated protein kinase (AMPK) pool or by its lipid composition (Chowdhury et al., 2026; Jain and Zoncu, 2022; Udayar et al., 2022; Zhang et al., 2021). Cholesterol accumulation in lysosomes leads to aberrant cholesterol mTORC1 signaling, impaired lysosomal proteolysis and mitophagy, resulting in aberrant mitochondrial morphology and function (Jain and Zoncu, 2022).

Besides MCSs, the mitochondria and the lysosome communicate largely through MDVs (Barazzuol et al., 2021; Fernandes et al., 2023). Degradative MDVs selectively transport oxidized mitochondrial cargo to lysosomes for degradation, whereas signaling MDVs traffic mitochondrial components through sorting endolysosomal sub compartments to drive innate and adaptive immune processes (Barazzuol et al., 2021; Fernandes et al., 2023). In addition, a subset of MDVs is targeted to peroxisomes, contributing to peroxisomal homeostasis and *de novo* biogenesis. The PD-related PTEN-induced kinase 1 (PINK1)/Parkin axis plays a central role in regulating MDVs trafficking by promoting their delivery to phagosomes and degradative lysosomes while preventing their incorporation into multivesicular bodies. This mechanism limits extracellular release of mitochondrial damage associated patterns (DAMPs) thereby restraining inflammation (Barazzuol et al., 2021; Fernandes et al., 2023; Ávila et al., 2025; Jain and Zoncu, 2022; van der Vlag et al., 2020). Accordingly, loss-of-function mutations in PINK1 or Parkin impair MDV trafficking, enhance mtDAMP release and neuroinflammation, and are associated with dopaminergic neuronal dysfunction and worsening motor symptoms in PD (Borsche et al., 2020; van der Vlag et al., 2020).

Lysosome-bound MDVs are generated in basal conditions but, when oxidative stress is increased, their formation is augmented. This usually leads to an increase in mitophagy (i.e., selective autophagic elimination of mitochondria) to restore proteostasis. Whereas the induction of nonselective macroautophagy promotes widespread autophagosome formation and destruction of cytoplasmic cargo, mitophagy involves local phagophore nucleation on the organelle and only delivery of mitochondria to the degradative endolysosome. The PD-associated PINK1/Parkin pathway is also a central regulator of mitophagy, while mutations in another PD-associated gene, LRRK2, have also been implicated in the dysregulation of this process (Borsche et al., 2020; Markaki and Tavernarakis, 2020; Picca et al., 2020). Defects in mitophagy have also been observed in AD-postmortem brains which has been proposed to be associated with an accumulation of dysfunctional mitochondria. Consequently, defective mitophagy has been recognized as a key contributor to PD and AD pathogenesis. Defects in mitophagy have also been associated with HD, where mitophagy is reduced by around seventy percent in the brain of HD mice (Markaki and Tavernarakis, 2020; Picca et al., 2020; Trigo et al., 2022; Zong et al., 2024). Thus, mitophagy is a tightly regulated inter-organelle system and age-dependent decline of mitophagy results in progressive mitochondrial accumulation and consequent deterioration of cell function. The cooperation between mitochondria and peroxisomes, although understudied, is also well recognized to have a major role in lipid metabolism and redox homeostasis (Barazzuol et al., 2021; Fernandes et al., 2023). During aging, both organelles are progressively impaired, resulting in reduced fatty acid oxidation capacity and oxidative stress, ultimately contributing for neurodegenerative processes (Barazzuol et al., 2021).

Despite increased evidence that inter-organelle communication is essential for cell homeostasis, current therapeutic strategies for NDs have largely focused on targeting individual molecular pathways. While these approaches have provided valuable mechanistic insights, their clinical efficacy has remained limited, likely because they fail to address the highly interconnected nature of cellular homeostasis. As we have been examining, organelles operate as components of an integrated signaling network in which dysfunction in one compartment rapidly propagates to others, creating self-amplifying cycles of bioenergetic failure, proteostasis collapse, impaired autophagy, and inflammatory activation. Consequently, interventions directed at a single pathway may transiently alleviate one aspect of the pathology while leaving the underlying network instability unresolved. Restoring inter-organelle communication, rather than correcting isolated defects, therefore represents a more comprehensive strategy to re-establish cellular equilibrium and improve the ability of cells to adapt to stress. This systems-level perspective also redefines the goals of regenerative medicine. In this emerging paradigm, regeneration is no longer defined solely by the generation of new cells but by the restoration of the cellular networks that preserve tissue function under chronic stress. Such a shift from cell replacement to cellular resilience may represent one of the most promising directions for the next-generation of regenerative medicine, particularly for complex disorders such as neurodegenerative diseases, where widespread dysfunction precedes irreversible cell loss.

## 4 Cell-free regenerative therapies as multi-target modulators of organelle homeostasis

Given the heterogeneous and multifactorial nature of NDs, the development of effective disease-modifying therapies will likely rely on combined or multitarget approaches that can tackle several aspects of cellular dysfunction. Considerable progress has been made towards the development of stem cell-based therapies, which usually focus on replacing affected cell populations with exogenous healthy counterparts and/or stimulating reparative responses in endogenous cells (Hoang et al., 2022). While it has been postulated that transplanted stem cells induce tissue repair by several mechanisms, including their ability to engraft into the injury site and differentiate into specialized cell types, their regenerative actions seem to be largely mediated by the secretion of bioactive factors (Joshi et al., 2025; Pinho et al., 2020; Chimenti et al., 2010; Gnecchi et al., 2005). This cocktail of soluble biochemical mediators and EVs, termed *secretome*, can regulate tissue function and repair *via* paracrine signaling, making it possible to harness the natural secretory ability of stem cells to develop potential therapies for regenerative medicine (Daneshmandi et al., 2020).

Mesenchymal stem cells (MSCs) constitute one of the most common cell sources of therapeutic secretome across various fields of regenerative medicine, owing to their widespread availability in the human body and well-characterized anti-inflammatory, immunomodulatory, proangiogenic, and antiapoptotic properties (Daneshmandi et al., 2020). In the context of neurological disorders, the secretomes of neural stem cells (NSCs) and Schwann cells have also been explored (Karimian et al., 2024; Marques et al., 2018), although to a lower extent. These cell-free therapies provide a complex combination of trophic, immunomodulatory, and regulatory signals without requiring long-term survival, engraftment, or differentiation of transplanted cells, thereby potentially reducing concerns associated with cellular persistence, uncontrolled proliferation, and ectopic differentiation (Alpeeva et al., 2026; Chouaib et al., 2023; da Silva et al., 2023; Foo et al., 2021; Giovannelli et al., 2023). As such, compared with whole-cell/cell replacement approaches, secretome-based therapeutic strategies are particularly attractive because of their improved safety profiles, as well as increased scalability, cost-effectiveness and long-term storage stability. These therapies are also associated with high tunability, since secretome contents can be modulated to respond to specific biological needs (Vizoso et al., 2017). Additionally, secretome-based treatments can be dosed and administered in a highly controlled manner, similarly to traditional pharmaceutical agents (Vizoso et al., 2017; Maguire, 2013).

In broad terms, the secretome is composed of growth and differentiation factors, cytokines, chemokines, metabolites, nucleic acids, lipids, and other bioactive elements that can simultaneously target multiple biological processes, together with EVs that help deliver specific cargo. As such, secretome-based approaches tend not to target a single pathway or pathological mechanism, acting on whole biochemical networks instead. This makes them especially promising for pathological conditions that arise from the dysfunction of multiple homeostatic mechanisms, modulating cellular responses, such as inflammation, cell survival, and extracellular matrix remodeling, that are an integral part of tissue maintenance and repair throughout the body (Negrete-Diaz et al., 2026).

Although NDs are often highly heterogeneous in terms of etiology, pathophysiology and manifestation, they usually share a few key hallmarks. These include, as previously described, neuroinflammation, impaired autophagy and proteostasis, oxidative stress, and the disruption of the blood-brain barrier, all of which can potentially be targeted by secretome therapies (Negrete-Diaz et al., 2026). Analyses of the MSC secretome have shown the presence of brain derived-neurotrophic factor (BDNF), glial cell line-derived neurotrophic factor (GDNF), nerve growth factor (NGF), and other neurotrophic molecules that regulate neuronal survival and function (Marote et al., 2023; Marques et al., 2021; Marques et al., 2024). The MSC secretome can also target glial cells, shifting astrocytes and microglia towards less reactive, pro-regenerative phenotypes, which might be explained by the presence of anti-inflammatory cytokines (*e.g.*, interleukin (IL)-10, transforming growth factor (TGF)-β), miRNAs and other immunomodulatory mediators (Trabulo et al., 2026; Yao et al., 2026; Santamaria et al., 2021). Moreover, the MSC secretome seems to act on pathological protein aggregation on multiple fronts, coordinating the direct cleavage of protein aggregates by matrix metalloproteinases (Oh et al., 2017) and the modulation of autophagic-lysosomal systems for protein clearance (Han et al., 2022; Shin et al., 2014). Oxidative stress and mitochondrial dysfunction can also be targeted by secretome approaches, with a multifunctional mechanism of action that seems to include both the direct scavenging and the reduced formation of reactive species, together with a potentiation of endogenous antioxidant defenses (Stavely and Nurgali, 2020).

This evidence supports the development of cell-free, secretome-based strategies as multifaceted approaches for the treatment of neurodegenerative disorders, given their potential to coordinate tissue repair and modulating functional cellular networks by acting on multiple therapeutic targets. Accordingly, the secretome of MSCs has consistently shown positive effects in models of AD, PD, HD, ALS and multiple sclerosis (MS), as well as in acute neurological conditions such as spinal cord injury (SCI) and acute ischemic stroke recently reviewed by (Giovannelli et al., 2023; Silva et al., 2023), which we will be describing in the next section.

## 5 Restoration of organelle homeostasis by stem cell-derived therapies

Recent studies have increasingly shown that the therapeutic benefits of stem cell-derived therapies extend far beyond tissue repair. Through the coordinated modulation of multiple organelle-associated pathways, including mitochondrial function, endoplasmic reticulum stress, autophagy-lysosomal activity, redox balance, and inflammatory signaling, these therapies promote the restoration of intracellular homeostasis and enhance the resilience of surviving cells. In this section, we will provide a brief overview of recent findings on the effects of stem cell-derived therapies in NDs and reflect on key aspects that still require further consideration and investigation (Figure 2).

**FIGURE 2:**
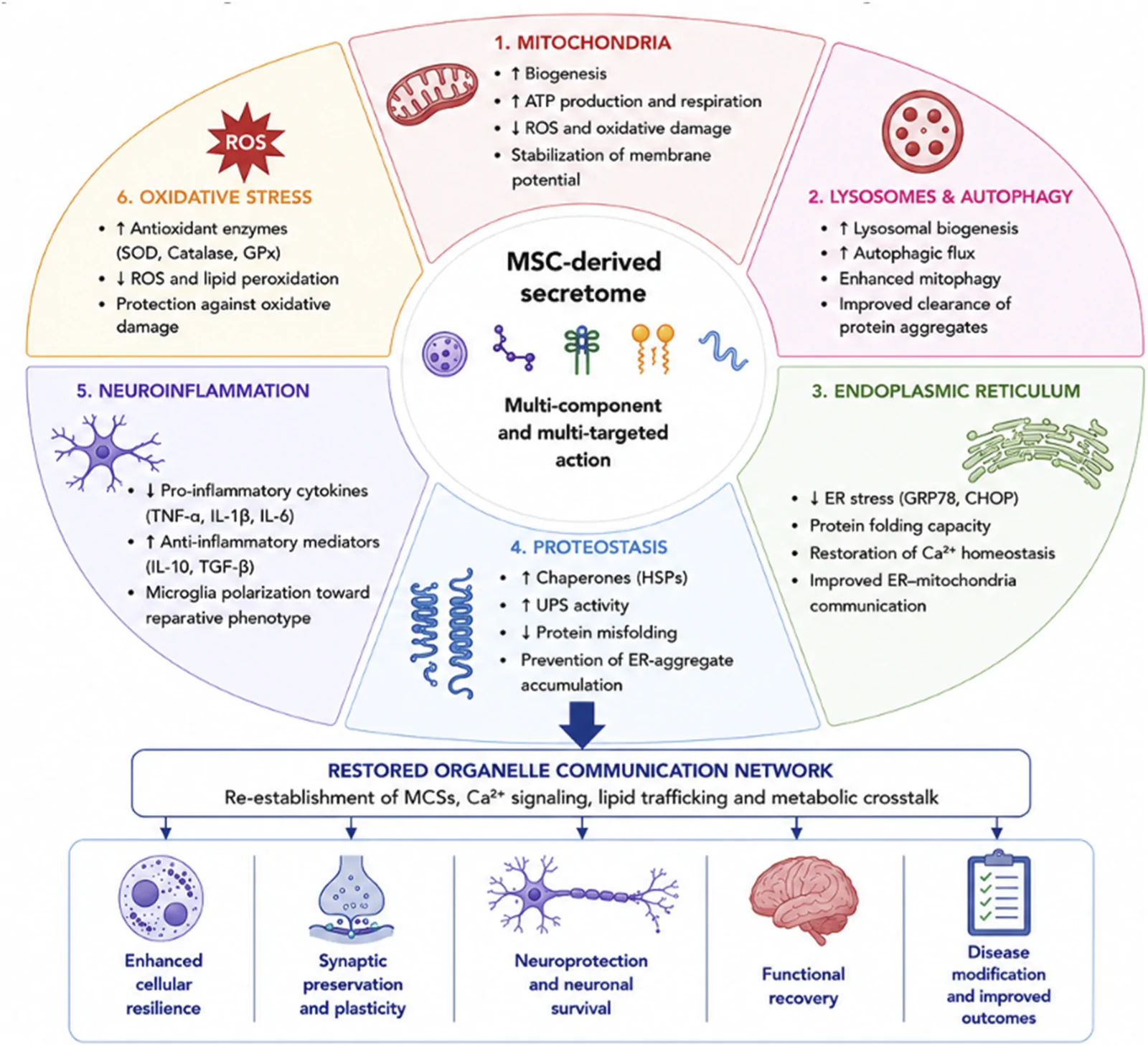
Mesenchymal stem cell (MSC)-derived secretome restores organelle homeostasis through the coordinated modulation of interconnected cellular pathways. The MSC-derived secretome, including extracellular vesicles (EVs), proteins, cytokines, lipids, metabolites and regulatory RNAs, simultaneously targets multiple organelle-associated processes rather than individual pathological mechanisms. These include mitochondrial function and bioenergetics, endoplasmic reticulum (ER) homeostasis, lysosomal function and autophagy, proteostasis, oxidative stress and neuroinflammation. The coordinated modulation of these interconnected pathways promotes the restoration of organelle communication networks, improving calcium (Ca^2+^) signaling, vesicular trafficking, lipid exchange and metabolic crosstalk. Ultimately, restoration of organelle homeostasis enhances cellular resilience, preserves synaptic function, promotes neuronal survival and neuroprotection, supports functional recovery, and may contribute to disease modification in neurodegenerative diseases. Created with BioRender.com.

Given the central role of mitochondrial dysfunction in neurodegeneration, numerous studies have investigated the ability of stem cell-derived therapies to improve mitochondrial homeostasis. These therapies have been shown to preserve mitochondrial dynamics, promote mitophagy, improve ATP production, reduce ROS accumulation, and limit apoptosis, thereby enhancing neuronal survival (Markaki and Tavernarakis, 2020; Moradi Vastegani et al., 2023; Trigo et al., 2022; Zong et al., 2024; Markaki and Tavernarakis, 2020; Picca et al., 2020; Trigo et al., 2022). A growing number of studies demonstrate that MSC-derived secretomes and EVs exert robust effects on mitochondrial quality control by simultaneously enhancing mitochondrial respiration, restoring membrane potential, promoting ATP synthesis, and reducing oxidative stress (Caplan and Dennis, 2006; da Silva et al., 2023; Drago et al., 2013; Fan et al., 2020; Fitzsimmons et al., 2018; Stavely and Nurgali, 2020). Beyond improving mitochondrial function, MSC-derived EVs have also been shown to activate pathways involved in mitochondrial turnover, including the PINK1/Parkin axis, AMPK signaling, and autophagy-related proteins such as Beclin-1 and LC3 (Escrig et al., 2019; Kaur et al., 2019; Carneiro-Pereira et al., 2025; Shin et al., 2014). These effects may promote the selective elimination of damaged mitochondria while facilitating mitochondrial renewal and metabolic adaptation. Importantly, preservation of mitochondrial quality has consequences that extend beyond the organelle itself. Functional mitochondria support ER calcium buffering, maintain lysosomal activity through adequate ATP production, and reduce inflammatory signaling derived from DAMPs. Consequently, the therapeutic benefits of MSC-derived secretomes likely arise not only from improving mitochondrial bioenergetics but also from restoring the central role of mitochondria within the intracellular organelle network. Importantly, restoration of mitochondrial quality control not only improves mitochondrial function but also preserves downstream communication with lysosomes and the ER.

As mentioned, disruption of ER–mitochondria communication has emerged as a common pathological feature across NDs, contributing to chronic ER stress, calcium dysregulation, mitochondrial dysfunction, and neuronal loss. Interestingly, increasing evidence indicates that MSC-derived secretomes and EVs can simultaneously modulate several of these interconnected processes. Some studies have demonstrated that MSC-derived products reduce ER stress by attenuating activation of the UPR while preserving mitochondrial membrane potential, ATP production, and oxidative phosphorylation (Assunção Silva et al., 2022; Bagno et al., 2018; Caplan and Dennis, 2006; da Silva et al., 2023; Drago et al., 2013; Fan et al., 2020; Fitzsimmons et al., 2018; Han et al., 2019; Han et al., 2022; Karimian et al., 2024; Trabulo et al., 2026; Tran and Damaser, 2015; Trigo et al., 2025; Turano et al., 2023; Vizoso et al., 2017). Growth factors such as HGF, BDNF, IGF-1 and VEGF, together with EV-associated miRNAs, have been implicated in reducing apoptosis, restoring intracellular calcium homeostasis, and promoting mitochondrial survival under pathological conditions (Karimian et al., 2024; Marote et al., 2016; Marote et al., 2023; Pinho et al., 2022; Pires et al., 2016). Although direct evidence demonstrating restoration of MAM architecture following MSC-secretome administration remains limited, the simultaneous improvement of ER function and mitochondrial bioenergetics strongly suggests that these therapies act upstream of individual stress pathways by preserving ER–mitochondria communication. Such coordinated regulation is particularly relevant given that perturbations in calcium transfer and lipid metabolism between these organelles amplify oxidative stress and proteostatic failure during neurodegeneration. Therefore, rather than independently targeting ER stress or mitochondrial dysfunction, cell-free therapies may influence the functional integration of these two organelles, thereby re-establishing cellular homeostasis.

Efficient lysosomal degradation and autophagic flux are essential for maintaining neuronal proteostasis, particularly in post-mitotic cells that cannot dilute damaged proteins through cell division. Impairment of lysosomal function contributes directly to the accumulation of toxic protein aggregates, defective organelle turnover, and chronic cellular stress observed across neurodegenerative diseases. Recent studies have demonstrated that MSC-derived secretomes promote autophagy by increasing expression of Beclin-1 and LC3-II while reducing the accumulation of p62, suggesting restoration of autophagic flux rather than simply increasing autophagosome formation (Escrig et al., 2019; Kaur et al., 2019; Carneiro-Pereira et al., 2025; Shin et al., 2014). Several signaling pathways appear to mediate these effects, including AMPK activation, mTOR inhibition, and regulation of the lysosomal transcription factor TFEB, a master regulator of lysosomal biogenesis and autophagy. By restoring lysosomal degradative capacity, MSC-derived products may help facilitate the clearance of dysfunctional mitochondria, aggregated proteins, and damaged cellular components. Importantly, these processes reinforce the reciprocal communication between lysosomes and mitochondria, preventing the establishment of pathological feed-forward loops in which mitochondrial dysfunction impairs lysosomal activity and *vice versa*. Thus, enhancement of autophagic-lysosomal function represents another mechanism through which cell-free therapies promote restoration of organelle homeostasis.

Protein homeostasis relies on the coordinated activity of protein synthesis, molecular chaperones, the UPR, the ubiquitin-proteasome system, and the autophagy-lysosomal pathway (Santra et al., 2019). Rather than representing independent quality control systems, these mechanisms operate as an integrated proteostatic network that closely interacts with mitochondrial metabolism and ER function. In NDs, failure of this network leads to the progressive accumulation of misfolded proteins such as α-synuclein, amyloid-β, tau, and mutant SOD1, ultimately overwhelming neuronal stress adaptation mechanisms (Riva et al., 2024; da Silva et al., 2023; Drago et al., 2013; Han et al., 2019; Hoang et al., 2022; Aranda-Abreu et al., 2026; Dugger and Dickson, 2017). MSC-derived secretomes have consistently demonstrated the ability to reduce pathological protein accumulation while enhancing endogenous mechanisms of protein quality control (Marques et al., 2021; Marques et al., 2024; Escrig et al., 2019; Feng et al., 2018; Kaniowska et al., 2022; Santamaria et al., 2021; Sha et al., 2021; Shin et al., 2014). Proteomic analyses have identified numerous chaperones, protein disulfide isomerases, protease inhibitors, and proteases within MSC secretomes that may contribute directly to protein folding and aggregate clearance (Marote et al., 2016; Marote et al., 2023; Marques et al., 2018; Vilaça-Faria et al., 2021). These findings suggest that cell-free therapies reinforce multiple arms of the proteostasis network simultaneously rather than targeting individual aggregation pathways.

Oxidative stress can be seen as both a consequence and a driver of organelle dysfunction. Excessive ROS production disrupts mitochondrial function, promotes ER stress, impairs lysosomal activity, and accelerates protein misfolding, thereby propagating dysfunction throughout the intracellular network. Consequently, restoration of redox homeostasis represents a critical step toward preserving organelle communication. MSC-derived secretomes and EVs contain several antioxidant enzymes, including catalase, as well as miRNAs and soluble factors capable of activating endogenous antioxidant pathways such as NRF2 signaling (Marote et al., 2016; Marote et al., 2023; Marques et al., 2018; Vilaça-Faria et al., 2021). These molecules have been shown to reduce intracellular ROS accumulation and to protect cells against oxidative stress damage (Ávila et al., 2025; Bonafede et al., 2016; He et al., 2023; Khan et al., 2017). Beyond limiting oxidative damage, improved redox homeostasis preserves mitochondrial function, supports efficient protein folding within the ER, and facilitates autophagic degradation (Ávila et al., 2025; Bonafede et al., 2016; He et al., 2023; Khan et al., 2017). Therefore, antioxidant effects should not be viewed as isolated therapeutic outcomes but rather as fundamental mechanisms contributing to restoration of intracellular organelle homeostasis.

Persistent neuroinflammation profoundly influences organelle function (Chowdhury et al., 2026; Jain and Zoncu, 2022; Wang and Yang, 2024). Pro-inflammatory cytokines released by activated microglia and astrocytes increase mitochondrial ROS production, induce ER stress, impair lysosomal degradation, and suppress autophagy, thereby exacerbating neuronal dysfunction (Aranda-Abreu et al., 2026; Picca et al., 2020; Teleanu et al., 2022). Conversely, mitochondrial damage further amplifies inflammatory responses through the release of DAMPs and activation of innate immune signaling pathways, establishing a vicious cycle between inflammation and organelle dysfunction. MSC-derived secretomes exert potent immunomodulatory effects through the delivery of anti-inflammatory cytokines, growth factors, and regulatory miRNAs that promote the transition of microglia toward neuroprotective phenotypes while reducing astrocyte reactivity (Giunti et al., 2021; González-Cubero et al., 2022; Harrell et al., 2019; Kaniowska et al., 2022; Kim et al., 2009). By attenuating inflammatory signaling, these therapies indirectly relieve chronic stress imposed on intracellular organelles, allowing restoration of mitochondrial metabolism, autophagic flux, and proteostasis. Thus, immune modulation represents an important upstream mechanism through which cell-free therapies promote recovery of the intracellular communication network rather than acting solely on immune cells.

Although most studies describe improvements in mitochondrial function, autophagy, ER stress, oxidative stress, or neuroinflammation independently, these observations likely represent complementary actions of different molecules present in MSC-derived secretome and EVs. Collectively, these coordinated effects may re-establish the dynamic interactions required for cellular adaptation and survival. However, direct visualization of restored membrane contacts sites or organelle communication following treatment with MSC-derived secretomes remains scarce and represents an important area for future investigation. Nevertheless, the convergence of evidence across multiple biological systems strongly supports the hypothesis that the therapeutic efficacy of cell-free regenerative therapies may derive from a coordinated improvement of organelle homeostasis rather than to modulate isolated molecular pathways. This systems-level mechanism provides a conceptual framework that helps explain the remarkable pleiotropic effects consistently observed following MSC-derived secretome administration and positions organelle network restoration as a defining feature of next-generation regenerative medicine.

Nevertheless, most available studies rely on indirect functional readouts, including changes in mitochondrial membrane potential, ATP production, oxidative stress, ER-stress markers, calcium homeostasis, autophagic flux, or lysosomal activity. While coordinated changes across these pathways are consistent with improved inter-organelle communication, they do not by themselves demonstrate alterations in the physical architecture of membrane contact sites or in the molecular tethering complexes that establish them. To tackle this, further studies should focus on approaches capable of resolving organelle proximity and contact-site composition, such as *in situ* or electron microscopy, super-resolution imaging, genetically encoded membrane-contact-site reporters, FRET-based proximity sensors, or proximity-labeling proteomics targeting contact-site proteins. Such approaches could determine whether MSC secretome or extracellular vesicles directly alter the abundance, morphology, spatial distribution, or molecular composition of MAMs and other membrane contact sites. Therefore, the currently available evidence should be interpreted primarily as demonstrating improved functional coordination between organelles rather than definitive structural restoration of inter-organelle contact sites. Whether MSC-derived secretome effects involve direct remodeling of membrane contact sites, including MAMs, remains to be established and represents an important mechanistic direction for future investigation.

Additionally, the therapeutic relevance of MSC-derived secretome is both disease- and stage-dependent. In chronic neurodegenerative diseases such as AD, PD, HD, and ALS, secretome-mediated modulation of organelle homeostasis may be most relevant to preserving neurons and synaptic function that remain viable but are exposed to progressive cellular stress. Such an approach would be expected to have predominantly neuroprotective or disease-modifying effects rather than to reverse established neuronal loss. In contrast, in MS, the therapeutic objectives may differ according to the inflammatory or progressive phase of disease, with potential roles in immunomodulation, neuroprotection, and tissue repair. Importantly, these improvements in organelle homeostasis, neuronal resilience, or synaptic function should be considered intermediate therapeutic outcomes, and their clinical relevance must ultimately be established through corresponding improvements in disease-specific functional measures, such as cognitive or motor performance. On the other hand, acute neurological injuries present a distinct therapeutic context in which the timing of intervention is critical, as secretome-based approaches may target secondary injury during the acute or subacute phase and potentially support tissue remodeling during subsequent recovery. These distinctions in therapeutic approaches also suggest that MSC-derived secretome should not be considered a universal replacement for disease-specific therapies. Rather, its multimodal activity may allow it to complement existing treatments by targeting cellular stress and tissue responses that are not adequately addressed by therapies directed toward a single pathological pathway. In advanced NDs, where substantial neuronal loss has already occurred, combination with regenerative, cell-replacement, or rehabilitation-based approaches may be required to achieve meaningful functional recovery.

## 6 Towards the next-generation of cell-free regenerative medicine

Over the last few decades, considerable research efforts have focused on exploring the therapeutic potential of cell-free therapies (Alpeeva et al., 2026; Foo et al., 2021; Pinho et al., 2022; Carneiro-Pereira et al., 2025). Importantly, the cell secretome can be engineered and adapted to suit the specific requirements of each research project. It can be modified to target key pathological pathways, including hypoxia-induced mitochondrial dysfunction and ER stress inflammation mediated by cytokines, EVs carrying inflammatory signals to neighboring or distant cells and neurodegenerative processes associated with neuronal death (Seong et al., 2025; Ni et al., 2022; Zhou et al., 2024; da Silva et al., 2023; Monteiro et al., 2025; Műzes and Sipos, 2022).

Among the different components present in the secretome, EVs have gained significant attention as a potential therapeutic agent in regenerative medicine. These nanoparticles are secreted by every cell type. EVs are formed by a lipidic bilayer and carry bioactive molecules that have the capacity to modulate biological processes through intercellular communication (De Jong et al., 2014). Despite their crucial function, EVs present certain disadvantages that must be considered for clinical translation. These include limited drug-loading efficiency, rapid clearance, and challenges in manufacturing scale-up. To mitigate these problems, researchers started integrating EVs into biomaterials (Stiliyanov-Atanasov et al., 2025; Liang et al., 2025; Silva et al., 2023). In the domain of EVs, research is usually focused on modifying EVs to carry more cargo and improving targeting systems, by changing the outside layer of the EVs (Arifka et al., 2022; De Jong et al., 2014; Liang et al., 2025; Lu et al., 2022). The combination of biomaterials with EVs and secretome can vary and many researchers have employed cell-free therapy utilising hydrogels, scaffolds, and nanofibers (Arifka et al., 2022; Silva et al., 2023; Brennan et al., 2020).

To enhance the field of regenerative medicine and develop a new generation of therapies, it is imperative to adopt a holistic approach by incorporating omics-guided therapies into future therapeutic interventions. These strategies are employed to understand and identify new diseases by leveraging large-scale data from diverse biological levels, including DNA, RNA and proteins. This method helps unveil the underlying causes of disease and develop effective treatments (Ivanisevic and Sewduth, 2023; Lim et al., 2024). The omics-based approach can be combined with drug delivery and biomaterials design. The combination of these techniques is imperative to design a personalized therapy (Ivanisevic and Sewduth, 2023; Liu et al., 2026). In this manner, the therapy is tailored to address the specific needs of each person. However, given the complexity and the substantial extent of these data sets, it is important to employ advanced computational methodologies. The integration and interpretation of multi-omics data is enabled by artificial intelligence and machine learning, which facilitating the identification of therapeutic targets, the prediction of treatment outcomes, and the design of personalized cell-free regenerative medicine (Lin et al., 2025; Valous et al., 2024).

## 7 Translational challenges

Despite the enormous advances in next-generation cell-free regenerative medicine, it is still difficult to translate these new treatments to the clinic due to different challenges. To successfully translate technologies such as engineered secretomes, designed EVs, the use of biomaterials and even omics-guided therapeutic design, it is necessary to overcome scientific, technological and manufacturing barriers. It is important to address these challenges to make sure that these advanced therapies are safe, effective, reproducible and scalable.

Although the application of cell-based therapies has demonstrated significant potential in regenerative medicine, they also present several limitations, including the risk of immune rejection and donor- and manufacturing-related variables. Donor age and tissue source can affect MSC proliferative capacity, senescence, metabolic state, and paracrine activity, while passage number and culture conditions can further modify the secretome phenotype (Marote et al., 2023; Marques et al., 2018; Alpeeva et al., 2026). Nutrient availability, inflammatory stimulation, hypoxia or other forms of preconditioning, culture medium composition, and harvesting conditions may alter the abundance and composition of extracellular vesicles, proteins, lipids, metabolites, and nucleic acids (Carneiro-Pereira et al., 2025; Alpeeva et al., 2026). This may also influence the abundance of secretome components with potential relevance to organelle homeostasis, including regulatory RNAs, metabolites, mitochondrial-associated nucleic acids, and proteins, further contributing to functional variability between secretome preparations. Consequently, there is a growing need to develop alternative strategies that can overcome these challenges.

Another major translational challenge is the limited mechanistic understanding of how cell-free therapeutics work, as it is hard to identify the important active components, set the right doses, predict how well the treatment will work, and meet the requirements for it to be approved. Notwithstanding the encouraging outcomes observed in regenerative medicine studies that used secretome, the components of secretome that are exactly responsible for its therapeutic effect remain unknown. This complexity arises from the dynamic nature of secretome, which exhibits different responses according to the microenvironment changes (Daneshmandi et al., 2020).

The same limitations are applicable to EVs. To use this method in a clinical context, it is first necessary to understand the principal mediators of EVs cargo, which may comprise proteins, microRNAs, lipids and metabolites (Schank et al., 2026; Margolis and Sadovsky, 2019). Regenerative responses are mediated through multiple pathways in a simultaneous manner, such as inflammation modulation, mitochondrial rescue and immunomodulation. It is important to understand the relevance of each therapeutic modality in all these mechanisms prior to its translation into clinical practice. Another significant challenge in the clinical translation of secretome and EVs is the lack of standardized and validated potency assays. Unlike drugs, where the active component is a single molecule with a well-defined structure, secretome and EVs consist of a mixture of components. The way they work together is what makes them biologically active. Furthermore, the composition of secretome and EVs varies according to cell type, culture conditions, donor characteristics and isolation method (Alpeeva et al., 2026). Therefore, to ensure that these secretome and EV formulations are equivalent for utilization in a clinical context, it is necessary to develop a range of potency assay strategies.

Prior to the development of any translational product, it is imperative to undertake a comprehensive study of the biodistribution of cell-free therapeutic agents. This is essential to predict their therapeutic efficacy, safety, as well as the optimal administration route. Following the administration of EVs and secretome, a discrepancy in biofiltration profile is observed throughout the body. It was observed that the accumulation of EVs and secretome was subject to variation depending on the administration route (Schank et al., 2026; Gupta et al., 2023; Su et al., 2025). It has been demonstrated that, upon administration of cell-free therapies via intravenous injection, a greater accumulation of the components is observed in the liver, spleen, kidneys, and lungs (De Sousa et al., 2023; Gupta et al., 2023; Su et al., 2025). When the administration is oral or via subcutaneous injection, accumulation occurs mostly in the liver and in the gastrointestinal tract. Following intranasal administration, the biodistribution of the substances is predominantly in the brain. Nonetheless, the delivery of cell-free therapeutics to the CNS remains one of the greatest challenges in regenerative medicine. As demonstrated by previous studies, intranasal administration in conjunction with secretomes and EVs has been shown to enhance brain delivery (Herman et al., 2021; Su et al., 2025). The primary impediment to the success of cell-free therapies in reaching the CNS is their ability to cross the blood-brain barrier. To achieve the desired therapeutic outcomes, it is important to develop a target delivery system that will enhance CNS accumulation while concomitantly minimizing off-target distribution. Several promising approaches have been developed, including the engineering of secretome and EVs surfaces with the best administration route for the purpose of tackling the CNS (Alpeeva et al., 2026; Baldari et al., 2017; Da Silva et al., 2025; De Jong et al., 2014; De Sousa et al., 2023).

Therefore, the next-generation of cell-free regenerative medicine represents a substantial modification to the field of regenerative therapies, deviating from conventional methods of cell transplantation towards specially designed, customisable cell-free treatments. There have been lots of advances in secretome engineering, designer EVs, biomaterial-assisted delivery, omics-guided therapeutic design, and artificial intelligence. It is evident that these factors have resulted in a significant enhancement in the utility of cell-free approaches for the promotion of tissue and cell repair, reverse damage, and functional recovery. However, for this to work in a clinical context, it will be necessary to overcome some challenges. These include a lack of understanding regarding how the therapeutics work, their large-scale manufacturing, their efficacy in the body, their biodistribution, how to deliver them to a specific target and the right doses, and getting official approval. To achieve these objectives, it is essential that research from diverse fields, including cell biology, bioengineering, medicine and computer science, collaborate effectively. This will ensure that these treatments are both safe and effective, and personalized to individual patients.

## 8 Conclusions and future perspectives

The understanding of NDs has undergone a profound transformation, shifting from a view centered on isolated pathological events toward the recognition that neuronal dysfunction arises from the progressive breakdown of an interconnected intracellular network. Rather than acting independently, the organelles continuously communicate to coordinate bioenergetics, proteostasis, calcium homeostasis, stress adaptation, and immune signaling. Consequently, disruption of one organelle rapidly propagates throughout the cellular network, amplifying dysfunction and ultimately compromising neuronal survival. This systems-level perspective suggests that effective regenerative strategies should move beyond correcting individual molecular defects and instead focus on restoring organelle homeostasis and the communication networks that maintain cellular resilience.

In this context, cell-free regenerative therapies, particularly MSC-derived secretomes and EVs, have emerged as promising therapeutic approaches. Their complex composition of growth factors, cytokines, lipids, metabolites, proteins, and regulatory RNAs enables the simultaneous modulation of multiple interconnected pathways, including mitochondrial function, endoplasmic reticulum stress, autophagy-lysosomal activity, oxidative stress, proteostasis, and neuroinflammation. Rather than targeting a single disease mechanism, stem cell-derived secretomes and EVs have the potential to re-establish intracellular homeostasis and enhance the adaptive capacity of surviving cells, offering a therapeutic strategy that more closely reflects the multifactorial nature of neurodegenerative diseases.

Although important challenges remain, including standardized manufacturing, potency assessment, biodistribution, delivery strategies, and mechanistic characterization, continued advances in multi-omics technologies, biomarker discovery, bioengineering, and artificial intelligence are expected to accelerate the development of next-generation cell-free therapeutics. Ultimately, targeting organelle homeostasis as a unifying principle for regenerative medicine may redefine how complex diseases are treated, shifting the field from replacing damaged cells toward restoring the cellular networks that preserve tissue function and resilience. While this concept has relevance for neurodegenerative disorders, its implications are likely to extend across a broad range of chronic diseases characterized by disrupted intracellular homeostasis, opening new avenues for precision regenerative medicine.
